# Sexual Health Education in Nursing: A Scoping Review Based on the Dialectical Structural Approach to Care in Spain

**DOI:** 10.3390/healthcare13151911

**Published:** 2025-08-05

**Authors:** Mónica Raquel Pereira-Afonso, Raquel Fernandez-Cézar, Victoria Lopezosa-Villajos, Miriam Hermida-Mota, Maria Angélica de Almeida Peres, Sagrario Gómez-Cantarino

**Affiliations:** 1 Faculty of Physiotherapy and Nursing, Toledo Campus, University of Castilla-La Mancha, s/n 45071, Av. de Carlos III, 45004 Toledo, Spain; monicaraquel.pereira@alu.uclm.es (M.R.P.-A.); miriam.hermida@alu.uclm.es (M.H.-M.); sagrario.gomez@uclm.es (S.G.-C.); 2 Critical Perspective Research Group, Department of Mathematics, Faculty of Education of Toledo, University of Castilla-La Mancha, s/n 45071, Av. de Carlos III, 45004 Toledo, Spain; raquel.fcezar@uclm.es; 3 Anna Nery School of Nursing, Federal University of Rio de Janeiro, Rio de Janeiro 21941-617, Brazil; mangelica.ufrj@gmail.com

**Keywords:** sexual education, health, nursing, sexual health, sexuality, STI prevention and management

## Abstract

Sexual health constitutes a fundamental aspect of overall well-being, with direct implications for individual development and the broader social and economic progress of communities. Promoting environments that ensure sexual experiences free from coercion, discrimination, and violence is a key public health priority. Sexuality, in this regard, should be understood as an inherent dimension of human experience, shaped by biological, cultural, cognitive, and ideological factors. Accordingly, sexual health education requires a holistic and multidimensional approach that integrates sociocultural, biographical, and professional perspectives. This study aims to examine the level of knowledge and training in sexual health among nursing students and healthcare professionals, as well as to assess the extent to which sexual health content is incorporated into nursing curricula at Spanish universities. A scoping review was conducted using the Dialectical Structural Model of Care (DSMC) as the theoretical framework. The findings indicate a significant lack of knowledge regarding sexual health among both nursing students and healthcare professionals, largely due to educational and structural limitations. Furthermore, sexual health education remains underrepresented in nursing curricula and is frequently addressed from a narrow, fragmented biomedical perspective. These results highlight the urgent need for the comprehensive integration of sexual health content into nursing education. Strengthening curricular inclusion is essential to ensure the preparation of competent professionals capable of delivering holistic, inclusive, and empowering care in this critical area of health.

## 1. Introduction

Sexual health constitutes a key pillar of overall well-being, influencing not only individual development but also the social and economic progress of communities and nations. In this regard, it is a public health priority to foster safe environments that support satisfying sexual experiences free from coercion, discrimination, or violence [1].

Sexuality, education, and the individual should be understood as interrelated dimensions, given that sexuality is an inherent aspect of human existence, shaped by biology, culture, knowledge, and personal beliefs [2]. While affective sexual education typically focuses on the emotional, affective, and relational aspects of sexuality, comprehensive sex education encompasses a broader scope that also includes prevention, sexual rights, diversity, and reproductive health. Therefore, sexual education must be approached from a holistic and multidimensional perspective, incorporating sociocultural, biographical, and professional elements [3].

However, despite the recognized importance of sexual health, there remain significant gaps and barriers in the current literature—particularly in how sexual health education is integrated into healthcare practice. These gaps include a lack of standardized content, insufficient emphasis on cultural sensitivity, and limited training opportunities for health professionals, especially nurses [3].

This underscores the need for a clearer understanding of how nursing education addresses sexual health and the competencies required to deliver effective, empathetic, and evidence-based care in this domain. It is essential to establish a stronger connection between sexual health education and nursing practice. Why is sexual health education vital for nurses? As frontline healthcare providers, nurses often serve as the first point of contact for individuals seeking guidance on sexual health issues. Their ability to provide accurate information, address misconceptions, and foster open, nonjudgmental dialogue is crucial to promoting patient well-being and reducing stigma [2,3].

A focused examination of the current educational strategies—and their limitations—can inform more comprehensive and contextually appropriate interventions within nursing curricula, ultimately strengthening the role of nurses in advancing sexual health at both individual and population levels.

In 1974, the World Health Organization (WHO) defined sexual health as a state of physical, emotional, mental, and social well-being in relation to sexuality, extending beyond the mere absence of disease [4]. This definition was later expanded at the 1994 International Conference on Population and Development, where reproductive health was recognized as a fundamental human right [5]. Since 2010, sexual health has been further consolidated as a comprehensive construct that encompasses not only physical and mental well-being, but also individuals’ overall quality of life [6].

This holistic conceptualization has significantly influenced international frameworks for health education, including the training of nursing professionals. Global organizations such as the WHO, UNESCO, and the European Union have developed guidelines advocating for the integration of sexual and reproductive health content into health sciences curricula [4]. Notably, in 2018, United Nations Educational, Scientific, and Cultural Organization (UNESCO), together with other United Nations agencies, published the International Technical Guidance on Sexuality Education, which emphasizes the importance of preparing healthcare professionals to address issues related to human sexuality in an ethical, evidence-based, and culturally sensitive manner [6,7].

Education and training are fundamental to the implementation of effective sexual health strategies. Populations require accurate, evidence-based, and age-appropriate information on the physical, psychological, and social dimensions of sexuality and reproduction [7]. Comprehensive sexual education contributes to disease prevention, the correction of misconceptions, and the promotion of safe sexual behaviors. Although primary care settings play a key role in delivering this education, its initiation should begin in early schooling and continue through higher education [8,9].

Within educational frameworks, sexually transmitted infections (STIs) remain a critical priority due to their potential to cause serious health outcomes, including infectious syndromes, immune compromise, and certain cancers [9]. Current global estimates indicate over one million new STI cases occur daily. Annually, approximately 374 million new cases of chlamydia, gonorrhea, syphilis, and trichomoniasis are reported; 500 million individuals are affected by *herpes simplex virus; and human papillomavirus (HPV*) accounts for around 300,000 cervical cancer deaths. Moreover, *hepatitis B* affects roughly 300 million people worldwide. In 2021, 38.4 million individuals were living with *HIV*, with 650,000 AIDS-related deaths reported [10,11].

In Europe, the European Centre for Disease Prevention and Control (ECDC) has coordinated epidemiological surveillance of sexually transmitted infections (STIs) since 2009. In Spain, this surveillance is carried out through the Compulsory Notifiable Disease System (EDO) and the Microbiological Information System of the National Epidemiological Surveillance Network (RENAVE), as established by Royal Decree 2210/1995. This legislation mandates the reporting of notifiable infections, including *hepatitis B*, *Chlamydia trachomatis*, *gonorrhea*, *syphilis* (including *congenital syphilis*), *lymphogranuloma venereum*, and *HIV/AIDS* [12,13,14].

Epidemiological data from 2021 reveal a persistently high incidence of STIs in Spain. Reported cases included 15,338 of gonorrhea (83.3% in men, primarily aged 25–34), with 231 *HIV* coinfections and 6952 coinfections with other STIs; 6613 of *syphilis* (90.5% in men aged 25–44), including 118 coinfections and 5 congenital cases; and 20,507 of *Chlamydia trachomatis* infection, with near-equal distribution between sexes and the highest prevalence in the 25–34 age group. Additionally, 649 cases of *lymphogranuloma venereum* were recorded, 98.8% in men. These figures underscore the urgent need to reinforce sexual health education and prevention strategies, particularly among young adults.

This study aims to analyze nursing students’ knowledge of sexual health through a review of the current scientific literature and an examination of sexual health content within Spanish university curricula. It also evaluates the knowledge, attitudes, and competencies of healthcare professionals, recognizing their dual role in clinical care and health education. Three hypotheses guide the research: (1) Nursing students have significant knowledge gaps regarding sexual health and STI prevention. (2) University curricula address these topics inconsistently, leading to educational disparities. (3) Healthcare professionals often feel unprepared to address sexuality-related issues, which hinders their educational and clinical effectiveness.

From the holistic paradigm of nursing, the human being is conceived of as an interconnected whole, in which the biological, psychological, emotional, spiritual, and social dimensions influence each other. This approach recognizes that health is not merely the absence of disease, but a dynamic state of balance and overall well-being. In this context, sexuality cannot be treated as an isolated aspect, but rather as an inseparable part of humanized care that requires understanding, sensitivity, and professional competence.

In this study, the dialectical model of care (SDMC) has been adopted. Although it has its own distinct characteristics, it is fully aligned with the holistic perspective. This model is based on the premise that care must be understood as a relational, dynamic, and situated practice, constructed through the interaction between caregivers and those being cared for. It is not about applying standardized protocols, but about addressing the real, contextualized, and evolving needs of individuals, taking into account their history, culture, embodiment, emotions, and social environment [15].

The SDMC assumes that care is an open, non-linear process in which tensions, contradictions, and ongoing learning coexist. This view is consistent with the philosophical foundations of holistic nursing, which acknowledge the complexity and uniqueness of each human being. Furthermore, the adoption of the dialectical model strengthens the active role of nurses as reflective care agents, capable of questioning their own practices, recognizing the social determinants of health, and adapting their interventions to the diverse life situations people experience. In the specific case of sexual health, this approach enables a more human, dialogical, and context-sensitive form of care [15,16].

The main conclusions highlight significant shortcomings in university-level sexual health education, both in terms of content coverage and pedagogical approach. Additionally, healthcare professionals report feeling insufficiently prepared to address sexual health issues in clinical settings, potentially undermining the quality of care delivered. A clear lack of consistency and integration of a holistic, interdisciplinary perspective on sexuality within academic programs is also evident. In this context, enhancing sexual health education—both at the undergraduate level and through ongoing professional development—emerges as a critical strategy to reduce STI incidence, encourage healthy sexual behaviors, and improve population-wide well-being.

## 2. Materials and Methods

### 2.1. Study Design

This article employed a scoping review as the methodological approach to address the study’s objectives. The review aimed to explore the relationship between nursing students’ and healthcare professionals’ knowledge of sexual health and the incorporation of sexual education within university nursing curricula in Spain. Scoping reviews are particularly suitable for mapping the scoping of a body of literature on a specific topic and identifying key concepts, knowledge gaps, and types of evidence available [15,16].

The SDMC [16,17] was applied due to its capacity to delve into the cultural and social foundations of structures, particularly those related to sexual diversity. This model identifies the functional dynamics of structures, allowing for the analysis of underlying causes that drive their transformation [15,17].

The SDMC methodology is grounded in a set of interrelated structures that support the processes of data management and analysis [17]. Its application in this study is especially relevant given the focus on sociocultural aspects of care. The model comprises three interdependent components, enabling a comprehensive analysis of phenomena from the perspective of cultural history [18]. Functional Unit (FU): This refers to the basic social structure of coexistence and socialization, through which values, beliefs, knowledge, and emotions are transmitted. It forms the foundation for social, political, and scientific systems that support nursing practice. Within this context, nursing students are positioned as key actors in the construction of critical and ethical knowledge. This perspective not only reinforces the development of nursing as an evidence-based discipline but also highlights the importance of professional reflection in the education of future healthcare providers.

Functional Framework (FF): This component refers to the physical and institutional spaces where nursing activities take place. In the context of higher education, this corresponds to academic institutions where future health professionals are trained. Functional Element (FE): This includes the social actors responsible for managing and delivering care. In the academic domain, this primarily includes faculty members within university programs, as well as practicing healthcare professionals engaged in educational roles. These individuals lead the educational process, guiding students through their learning and professional development.

**Figure 1:**
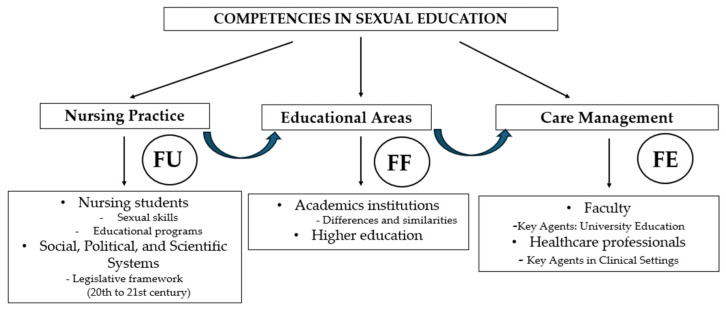
Structural dialectical model of care (DSMC): application of its frameworks. Source: Authors’ own elaboration.

The logical interconnection of these three components provides an effective analytical tool for organizing and examining data, thereby facilitating a holistic understanding of nursing as both a scientific and humanistic discipline (Figure 1).

The selection of the DSMC as the theoretical framework for this study responds to the need to understand sexual health education in nursing from an integrative, structural, and critical perspective. Unlike other models that approach health education or nursing practice through fragmented lenses, whether solely pedagogical, biomedical, or behavioral, the DSMC provides an analytical structure that allows for a systemic and dialectical interpretation of the complexity inherent in educational phenomena.

Within this framework, the Functional Unit (FU) enables the analysis of how training programs, sexual health policies, and legislative frameworks directly influence the construction of nursing students’ professional roles. At the same time, the Functional Factor (FF) highlights the influence of academic institutions in shaping curricular content and reproducing institutional values related to sexuality. Finally, the Structuring Factor (SF) allows for an understanding of how specific competencies are consolidated through pedagogical practices carried out by faculty members, and how these practices impact the comprehensive training of future nursing professionals. The use of the DSMC is justified not only for its explanatory capacity but also for its epistemological coherence with the objectives of this research, as it facilitates a contextualized, critical, and situated approach to sexual health within higher education in nursing.

### 2.2. Search Strategy

The scoping review process begins with an exploratory research question [19] aimed, in this case, at systematically synthesizing and critically analyzing existing knowledge [20]. In this case, “What is the knowledge of sexual health possessed by health agents?”.

This review included several steps [20]. Initially, the nursing student population was identified as the subject, and a research question was formulated: “Are the subjects included in the university curriculum sufficient to educate future health professionals about sexuality?” A qualitative documentary methodology was employed to analyze university nursing curricula, guided by the principles of a scoping review. The objective was to identify how sexual health education is integrated into nursing degree programs. Academic curricula from various universities were analyzed, taking into account the heterogeneity in course titles, formats, and structures. To ensure a rigorous systematization, a mixed coding framework was developed, combining both deductive and inductive strategies. In the deductive phase, an analytical matrix was structured based on the Dialectical Structural Method of Care (DSMC), specifically incorporating the components of the FU, FF, and FE. These theoretical axes informed the initial coding categories, including the presence of sexual health content, normative/ethical approach, stated competencies, role of faculty, and links to health or legislative policies.

In parallel, an inductive coding process was applied to the documentary corpus, allowing subcategories to emerge through interpretive reading of the curricular content. These included topics such as gender perspective, sexual diversity, thematic transversality, and employed pedagogical methodologies (Table 1). To ensure comparability and standardization, the data were harmonized using a technical data sheet containing variables such as course title, academic year, credit allocation, type of course (compulsory/elective), and mode of instruction. Data synthesis was conducted through thematic analysis, organizing categories in relation to the DSMC axes and their influence on the construction of the professional profile. To strengthen interpretative reliability, two researchers (M.R.P-A. and R.F-C.) independently conducted the initial data extraction. The third author (V.-L.-V.) reviewed and identified the key findings, while the fourth, fifth, and sixth authors (M.H-M., M.A.P., and SG-C.) delineated the common thematic lines, which were subsequently organized according to the structural components of the Dialectical Structural Method of Care (DSMC): the core (FU), the intermediate zone (FF), and the outer layer (FE). Discrepancies in the selection and inclusion of studies were resolved by consensus among the research team, ensuring methodological rigor and analytical consistency [17]. The researchers agreed on eligibility criteria, which had to include information about the education and knowledge of both university nursing students and faculty members and health professionals. The review covers documents analyzing the origin and evolution of sexuality and sexual education from a legislative perspective, including regulations such as Organic Law 1/2023 in Spain, which promotes training in sexual and reproductive health within the educational system [21]. Indeed, studies focused on nursing students are considered, evaluating the current state of the issue and university curricula [22,23]. Additionally, research examining the relationship of health professionals with sexual education is included, highlighting the need for specific training in this field.

Subsequently, a document search was conducted from October 2024 to April 2025. During this period, several databases were consulted: (1) PubMed, (2) Dialnet, (3) SciELO, (4) Cochrane, (5) Scopus. Following this search, physical and digital manuals were reviewed through the library services of the University of Castilla La Mancha. To obtain up-to-date information, the search was limited to the last 10 years (2015 to 2025). However, some publications prior to this period were also selected due to their relevance to this scoping review, including primary and legislative sources. For legislative sources, Official State Bulletins were consulted, among others. For database exploration, natural language or free text was used, standardized and controlled with MeSH and DeCS descriptors. These were combined with Boolean operators (“and/and”, “or/or”, “not/not”).

Moreover, combinations of words were used, when appropriate, to reflect the syntax and search rules common to individual databases. The descriptors used were health, education, sexuality, nursing student, sexually transmitted diseases, sexual health, and training programs.

### 2.3. Selection Process

**Figure 2:**
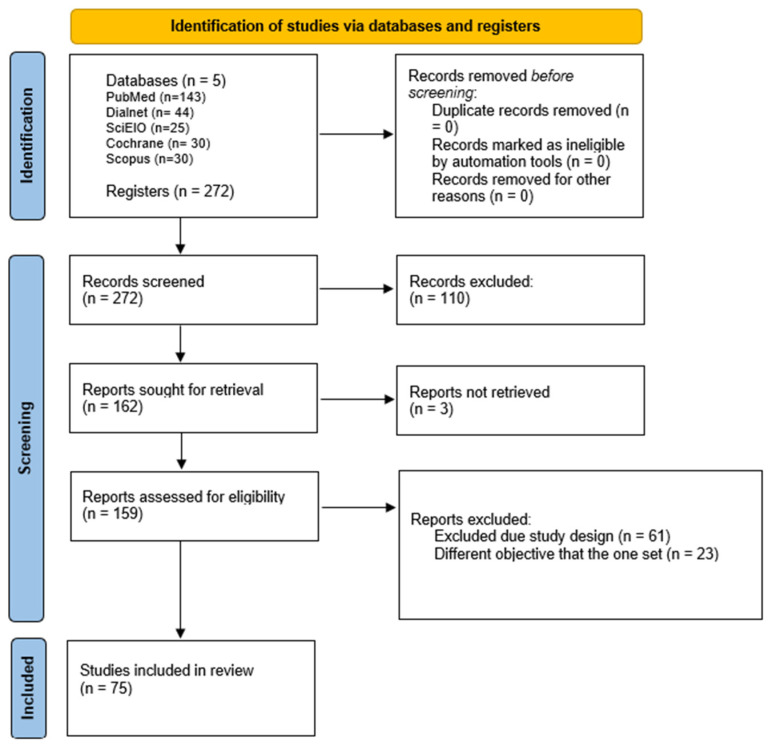
Flowchart for the study selection. This figure presents the databases used: PubMed, Dialnet, SciELO, Cochrane, and Scopus, from which a total of 272 articles were identified. After applying the inclusion and exclusion criteria, 110 documents were removed for not meeting the established requirements. Reasons for exclusion included: titles unrelated to the study topic, abstracts that did not address the research objective, non-relevant study populations, duplicate studies, and documents without access to the full text. Source: Author’s own elaboration.

This type of review aims to map the evidence supporting a particular research area and to identify gaps in the existing evidence [19]. This methodology does not intend to assess the methodological quality of the included studies nor to find the best scientific evidence, but rather to map the existing scientific evidence [20]. To ensure the relevance and quality of the sources selected for this scoping review, the following inclusion criteria were established: (1) documents that directly and explicitly address the central theme of the review, specifically those related to sexual health, sexual education, and nursing training programs; (2) original studies, systematic reviews, clinical guidelines, official reports, and scientific publications indexed in specialized journals and recognized databases were considered; (3) selection was preferably limited to documents published in the last ten years (2015–2025), with the exception of some historical and legislative sources deemed relevant to the context and understanding of the subject; (4) documents with full text available for detailed reading and analysis; (5) studies conducted in Spain or in contexts comparable at the university and healthcare levels, in order to maintain cultural and regulatory relevance to the study subject. A total of 75 documents met the requirements reflected in the inclusion and exclusion criteria. It is noteworthy that royal decrees, regulations, and legal provisions were consulted with the intention of specifying and enumerating some of their most relevant articles related to the study topic (Figure 2).

### 2.4. Content Analysis

The content analysis of the documentation was performed from a qualitative perspective through an objective and systematic approach [19]. The steps carried out for the analysis consisted of the following: (1) thematic linkage; (2) preliminary classification of the documents based on content and organizational criteria; (3) selection and extraction of relevant information according to the scoping review criteria, to allow results and conclusions [3,7,22].

The selected articles were analyzed from the perspective of the three thematic blocks studied, each encompassed within the structures forming the SDCM [17]: (1) nursing students; (2) university training programs; (3) health professionals.

To extract and summarize data, the researchers performed an inferential interpretation. The aim was to understand the already investigated and documented reality regarding the knowledge of current and future health professionals about sexuality. Studies chosen for analysis were not excluded based on their degree of rigor, since the objective of the scoping review was to synthesize the results of the reviewed research to extrapolate greater knowledge and insight into healthcare for the scientific community [17].

After a systematic process of reading and re-reading the selected articles, it was possible to answer the guiding question posed in this study: “What is the level of knowledge about sexual health possessed by health agents?” Additionally, it was analyzed whether the contents included in university curricula are sufficient to adequately train future health professionals in matters of sexuality.

## 3. Results

In all groups of living beings, except for the human species, sexuality is a vital event characterized by annual cycles and species-specific behaviors, such as courtship, mating, incubation, or differentiation between males and females [100,101]. However, in the human species, these components are no longer fundamental for survival; thus, without disregarding the perpetuation of the species, sexuality represents a turning point where personality, cultural creativity, and personal expression develop [102,103,104,105,106]. Consequently, all societies have developed accepted sexual norms that identify personalities and identities within the group, which entails an assignment of roles and functions within each community [103,104]. Therefore, sexuality can be classified into three blocks to differentiate sex, sexual identity, sexuality, and erotica: (1) the biology of sex, considering physiology, hormones, and neurological connections; (2) the cultural aspects in which sexual desire is formed and developed; (3) individual experiences and the ways in which people express their sexuality [104,105]. In the 1950s, the Kinsey Report was the first to analyze sexual behaviors in men and women, introducing the concept of sexual orientation as a physical response to erotic stimuli [107]. Masters and Johnson later described the human sexual response in four phases: excitement, plateau, orgasm, and resolution [103]. In the 1980s, authors such as Kaplan, LoPiccolo, and Carrobles [107] proposed alternative models focusing on desire, satisfaction, and perception. Currently, these theories are considered obsolete. Instead, a broader view is accepted: sexual desire is understood as an emotion influenced by biological, psychological, social, and cultural factors, as proposed by psychologist Javier Gómez Zapiain [108].

### 3.1. Sexual Competencies in Nursing: Training from the University Faculty and Its Relationship with Legislative, Social, and Scientific Systems

In 1978, the Spanish Constitution established in its Article 27 the right to education, recognizing the right to freedom of teaching [24]. This education should allow the development of each individual’s personality while respecting fundamental rights and including concepts such as affectivity, emotional intelligence, sexuality, and gender equality, among others [2,24].

Since 2003, sexual education has been mandatory in the curricula of 19 European countries. This education can be approached from three perspectives: (1) abstinence-centered; (2) abstinence combined with safe sex and contraceptive methods; (3) a broader view that incorporates the previous concepts and adds personal growth [2]. Currently, in Spain, there is no legal requirement to include sexual education in training programs. In the latest educational reform carried out in 2020 with Organic Law 3/2020, of December 29 (LOMLOE) [25], Organic Law 2/2006 of Education (LOE) [26] was modified, strengthening the inclusive, comprehensive, and transversal approach to sexual education within the school curriculum. Although it does not establish a specific subject for sexual education, it promotes its inclusion across various curricular areas [25].

**Figure 3:**
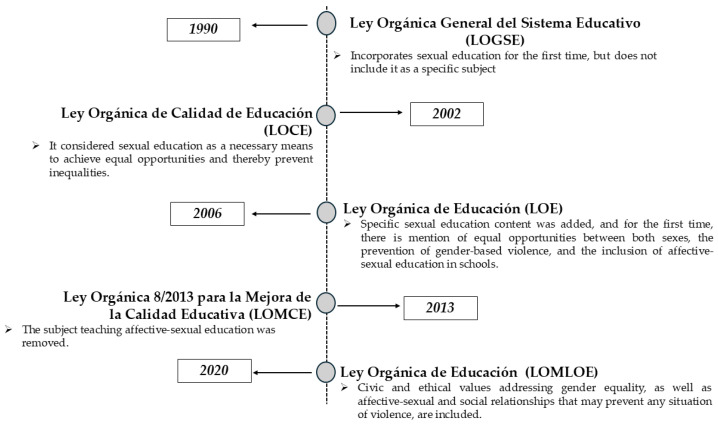
Evolution of sexual education in Spain through legislation. Source: Authors’ own elaboration.

Although sexual education in Spain has been included over the years (Figure 3), allowing students to acquire certain concepts of equality and respect, it does not guarantee comprehensive sexual education since it does not address the promotion of knowledge, skills, and attitudes that improve health and well-being [27,28], as advocated by the United Nations Educational, Scientific, and Cultural Organization (UNESCO) through comprehensive sexual education programs [100].

In the context of university education in Spain, the nursing degree is offered through curricula designed by each university. These programs must comply with the guidelines established in Royal Decree 822/2021, of September 28, which regulates the organization of university education and the procedure to ensure its quality [29]. Commonly, the nursing degree curriculum at the national level consists of a total of 240 credits, of which at least 60 are basic training credits in specific competencies (Table 2). It also comprises basic aspects of the Health Sciences branch, compulsory subjects, electives, seminars, external practicums, assignments, other training activities, and the preparation and defense of the final degree project required to obtain the qualification [29,30].

The following describes the sexuality-related content included in the training programs of the nursing degree at public universities in Spain. For this purpose, the autonomous community, the subject, the type of course (compulsory, basic, elective), and the related syllabus were taken into account. Spain is territorially organized into 17 autonomous communities (CCAA) and two autonomous cities, each with authority over higher education. Within this decentralized framework, each region hosts universities that, while aligned with national legal frameworks, retain autonomy to develop their own curricula and internal regulations. This structural diversity justifies the need for a disaggregated analysis by autonomous community, enabling the identification of both similarities and differences in how sexual health content is integrated into undergraduate nursing programs. To support this analysis, a comparative table was developed and organized by region, facilitating the systematic synthesis of data and the recognition of regional curricular patterns. This analytical approach aligns with the objective of highlighting the curricular diversity across Spanish territories and ensuring a context-sensitive interpretation of the findings (Table 3).

Analysis of the nursing degree curricula reveals that sexual education is present across academic programs; however, its depth, mandatory status, and pedagogical approach vary considerably among regions and universities [31,32,33,34,35,36,37,38,39,40,41,42,43,44,45,46,47,48,49,50,51,52,53,54,55,56,57,58,59,60,61,62,63,64,65,66,67,68,69,70,71,72,73,74,75]. Most courses addressing sexuality are compulsory, which in principle ensures a minimum standardized training in this area. Nevertheless, certain contents appear in basic or elective courses—albeit less frequently—highlighting differences in the curricular prioritization of the topic. From a territorial perspective, regions such as Andalusia [38,39,40,41,42,43,44,45], Catalonia [46,47,48,49,50], the Valencian Community [35,36,37], and Madrid [31,32,33,34] stand out, as multiple universities within them offer compulsory content related to sexual and reproductive health, family planning, sexually transmitted infections (STIs), fertility and infertility, and sexual dysfunctions. Other regions, such as Galicia [52,53,54,55], Castile and León [62,63,64,65], Navarre [58], the Basque Country [61], and Murcia [51], although with fewer institutions, also include relevant topics such as sexual education, gender, contraception, or adolescent sexuality.

The most recurrent topics identified in the curricula analyzed include (1) family planning and contraceptive methods, (2) sexually transmitted infections (STIs/STDs), (3) sexual and reproductive health, (4) infertility and reproductive cycle disorders, and (5) sexual education, gender, and sexual violence.

In addition, certain universities—particularly those in Extremadura [57], Cantabria [56,57,58], Castile-La Mancha [72,73,74,75], and the campuses of Ceuta [70] and Melilla [71]—approach sexuality from psychosocial and gender-based perspectives, incorporating issues such as sexual diversity, sexual violence, harassment, and sexual health in vulnerable populations.

Several factors may explain the observed disparities in curricular inclusion. Firstly, universities located in major urban centers with long-standing academic traditions—such as Madrid, Barcelona, Valencia, Seville, or Granada—tend to offer broader and more diverse sexuality-related content. These institutions may have greater access to specialized teaching staff and foster more open, interdisciplinary academic cultures that integrate health, sexual rights, gender perspectives, and diversity.

Conversely, in regions with lower population density or without large urban hubs—such as La Rioja, parts of Castile-La Mancha, or Extremadura—content tends to be more limited and focused on biomedical or preventive aspects, such as STIs or contraception, with less emphasis on sexual or social dimensions.

Moreover, regional public health policies on sexual and reproductive health play a crucial role [28]. Autonomous communities such as Catalonia, Andalusia, and the Valencian Community demonstrate a more comprehensive curricular integration of sexuality.

Universities with a stronger orientation toward public health, prevention, and the social determinants of health tend to offer more critical and comprehensive training in this area [7,8,9]. Others adopt more biomedical-centered models, often overlooking psycho-emotional or social dimensions essential for holistic sexual health care [27,28].

Although sexual health training is generally present in nursing degree programs across Spain, significant disparities remain at both regional and institutional levels. This heterogeneity underscores the need for greater curricular standardization, ensuring equitable, evidence-based, and rights-oriented education for all future nursing professionals. Promoting comprehensive sexual education within the university context not only enhances the quality of care but also supports ethical and humanized nursing practice [76].

### 3.2. Sexuality Education Within the Educational Framework: The Role of Academic Institutions in Nursing

Educational institutions offer an ideal setting for addressing specific health issues [76]. In Europe, individuals typically engage in formal education for more than ten years, a period that contributes significantly to personal and social development [77]. Numerous pedagogical studies highlight the effectiveness of educational settings in transmitting knowledge, fostering skills, and promoting healthy decision-making. Accordingly, sexual health promotion and prevention are considered essential components within these institutions [78,79,80].

The provision of accurate knowledge and appropriate access to sexual and reproductive health services for young populations is crucial in preventing and detecting serious health problems. Evidence from existing research demonstrates that comprehensive sex education programs are associated with delayed initiation of sexual activity—currently between ages 15 and 17—increased use of barrier methods, and greater contraceptive uptake [79,80]. The 2019 National Survey on Sexual Health and Contraception among Spanish Youth revealed considerable gaps in sexual and contraceptive education; 68.5% of participants reported not feeling adequately informed to address these issues [81].

It is important to consider that the university period is when most students begin exploring their intimate relationships and sexual identity. However, a lack of formal training often leaves them ill-equipped for informed decision-making [79]. Education for health sciences students—specifically in nursing—should be designed to address the normalization, understanding, and critical analysis of sexuality, incorporating students’ own experiences, attitudes, and beliefs [80]. Although senior students tend to demonstrate more informed sexual behaviors than undergraduates, faculty often prioritize content related to sexually transmitted infections (STIs) while neglecting broader sexual dimensions. This imbalance underscores the need for faculty to receive high-quality training in sexuality education [82].

Due to the identification of substantial knowledge gaps on sexual education within higher education institutions, various universities have conducted assessments and implemented research projects aimed at analyzing the current state of this topic [78]. At the University of Alicante, a study by the Educational Innovation Network for Psycho-Emotional Competence Learning found significant deficiencies in topics such as adolescent pregnancy (79%), STIs (78%), contraception (72%), and sexual anatomy and physiology (49%). Male students expressed greater uncertainty regarding STIs (84.1%), while female students were more concerned about adolescent pregnancy (84.1%) [78,82].

At the University of Palencia, a survey assessing nursing students’ contraceptive knowledge revealed that 24.2% reported doubts about the topic [83,84]. The condom was the most well-known method (100%). Despite 93.2% of participants acknowledging the ineffectiveness of the “withdrawal method,” 45.6% reported having used it. Only 28.6% of students reported having received formal training from health professionals [84].

Similarly, the University of Barcelona assessed nursing students’ attitudes toward *HIV/AIDS* using the Attitude Toward AIDS Scale validated by Tomás-Sábado [85]. The results showed that 20–25% of students were unaware of their obligation to maintain confidentiality for *HIV*-positive patients; 28% believed that caregivers should be warned before providing care to infected individuals; and up to 40% were unsure about *HIV* transmission routes. These findings highlight the negative attitudes and anxieties that students may experience when caring for *HIV*-positive patients—stemming from a lack of education focused on relevant knowledge and skills [85,86].

At the University of Ronda, a study conducted with 172 nursing students found that only 8.9% would consult a healthcare professional with sexual health questions, and only 26% had received any sexual health education at university [87]. When asked whether the withdrawal method was safe, 44.1% of those who answered incorrectly had received their sexual education in high school, and 26.1% during university. Furthermore, 10% of participants were unaware that condoms are the only method that protects against STIs [88].

A cross-sectional descriptive study at the University of Seville involving 291 first-year nursing students explored risky behaviors such as alcohol consumption. The results showed that 65% of female students and 55% of male students reported using condoms. However, 45% of male students and 47% of female students reported using the withdrawal method. Additionally, 32% of the female students had taken emergency contraception. A significant correlation was found between alcohol consumption and unprotected sex [88].

Given the centrality of patient, family, and community care in nursing, it is essential for students to adopt a positive and informed attitude toward sexuality. A team of experts from the University of Burgos and South Tyneside and Sunderland NHS Foundation Trust (UK) evaluated whether first- and final-year nursing students had received adequate sexual education. The results showed that 95.2% believed their training was insufficient. Among them, 58% had not used any protection during sexual activity, and only 15% had undergone STI testing after unprotected sex. Additionally, 77% had acquired their sexual knowledge informally and without scientific basis, and 73.26% did not feel competent in sexual health matters [82].

Finally, a multicenter study conducted across seven Spanish universities found that students who had not received formal instruction on sexuality within their curriculum had significantly lower knowledge levels. Many students relied on the internet for answers, whereas those who had been taught by healthcare professionals showed better knowledge and attitudes. Some students reported not using barrier methods due to either unexpected sexual encounters or decreased sexual pleasure when using condoms [89]. These findings underscore the importance of providing robust, evidence-based formal education on sexuality to prevent violence, unintended pregnancies, and STIs [79].

The Functional Framework (MF) for education in undergraduate nursing programs is materialized through the curricula and institutional structures in which students are trained as future healthcare professionals. However, numerous studies [76,77,78,79,80,81,82,83,84,85,86,87,88,89] reveal that sexuality education within these programs remains scarce, superficial, or insufficiently integrated. This limited approach has significant implications: students lack essential knowledge for STI prevention, hold stigmatizing misconceptions, and fail to develop the competencies necessary for the sensitive and safe management of patients with STIs [77,78,79,80,81,82,83]. These findings underscore the need to reevaluate and strengthen the MF, incorporating sexual health content from a scientific, ethical, and human rights-based perspective—ultimately contributing to more informed, inclusive, and prejudice-free nursing care.

### 3.3. Nursing Educators and Healthcare Professionals: Key Agents in University-Based Sexuality Education

Healthcare professionals frequently encounter challenges when addressing sexuality in clinical practice. These difficulties are primarily linked to various professional barriers: 79% of healthcare workers report not feeling adequately trained to discuss sexuality, 67% cite a lack of time, and 50% experience embarrassment or discomfort when addressing the topic [90].

A study conducted at the University of Alicante revealed that nursing professionals did not feel sufficiently trained in sexual and reproductive health to provide adequate education to nursing students [78]. Similarly, healthcare workers in Primary Care Centers in Barcelona acknowledged the importance of training in sexual health, yet expressed significant limitations in addressing specific topics, such as sexual dysfunction. Among nursing faculty, sexuality is often taught in a superficial manner, largely restricted to biomedical aspects [91,102].

Additionally, the absence of established protocols and a lack of clarity regarding who is responsible for providing sexual health services contribute to confusion. Religious beliefs and homophobic attitudes may also play a role in constraining care delivery [92]. Many students in the health sciences report not having received adequate sexual health education during their academic training, often limited to content from obstetrics and gynecology courses. As a result, many do not feel confident addressing these issues in clinical practice [90].

Interviews conducted in 2018 with health education faculty highlighted persistent barriers faced by healthcare professionals, including inadequate behaviors, lack of time, and limited knowledge regarding sexual health [93]. In response, some educators have adopted online tools and computer-assisted instruction to facilitate sexuality education and overcome these limitations. Social media platforms are also increasingly being used to promote sexual health [94].

Establishing a relationship of trust between instructors and students is crucial. Such trust fosters a learning environment characterized by openness and comfort, which is essential for discussing sensitive topics like sexual health [95,96].

A survey evaluating nursing staff’s knowledge about sexually transmitted infections (STIs) revealed a lack of consistency in understanding within healthcare teams. While 70% of respondents reported knowing the transmission routes of STIs, only 3% accurately identified risk behaviors [97]. Knowledge gaps regarding *HIV/AIDS* and condom use were also prevalent. The study concluded that there is an urgent need for continuous education and training programs in sexual health for nursing professionals [97,98].

Another study identified that the STIs least understood by nurses included *chlamydia*, *hepatitis B*, and *hepatitis C* [98,99]. Moreover, 16.2% of participants were unaware that *HIV/AIDS* could be transmitted during pregnancy and breastfeeding. There was also significant confusion about the incubation period and diagnostic timelines of the disease [99].

In light of these findings, the Functional Element (FE)—understood as the set of social actors responsible for care delivery—takes on a critical role in the academic training of nursing students. Within this context, both educators and healthcare professionals involved in clinical education are essential to ensuring comprehensive training. However, numerous studies [78,90,91,92,93,94,95,96,97,98,99] consistently report a concerning deficit in sexual and reproductive health knowledge among those charged with teaching this content. These shortcomings may negatively affect students’ ability to develop essential competencies, such as critical thinking, empathy, and communication skills, required for delivering effective sexual health education and care [82,87].

Therefore, enhancing the training of the FE through updated, evidence-based content with a gender-sensitive and human rights approach to sexual and reproductive health is not only necessary but also a key strategy. Doing so will strengthen the quality of education within nursing programs and support the development of a more equitable, respectful, and competent model of healthcare delivery.

## 4. Discussion

Sexual health is a fundamental component of individual well-being, as it significantly influences personality development and intersects with the cultural, biological, economic, and social aspects of a nation [109]. However, it remains a historically taboo subject, frequently overlooked in educational settings—both in schools and universities—as well as within family environments [102,107]. In this context, the creation of safe spaces is essential to foster healthy sexual experiences, free from risky behaviors, coercion, and violence [110].

Upon entering university, young people are exposed to new stimuli, including broadened social networks, lifestyle changes, and increased autonomy. These factors place university students at greater risk compared to other demographic groups. The lack of sexual health information provided by educators, coupled with the initiation of sexual activity, is associated with an increase in sexually transmitted infections (STIs) among this population [84,111]. A cross-sectional study conducted at the University of Jaén revealed that nursing students enrolled in the course *Child and Adolescent Nursing* primarily obtained their sexual health information from the internet. Additionally, 6.9% of respondents of respondents exhibited low knowledge levels regarding contraception, particularly hormonal methods [89].

The male condom remains the most widely known and accessible contraceptive method. Nonetheless, up to 40% of sexually active individuals report inconsistent or no condom use, with higher rates observed among those in same-sex relationships or long-term partnerships [79,89,111].

University students often underestimate their vulnerability to STIs. However, high rates of infections such as human papillomavirus (HPV), genital herpes, and gonorrhea have been reported within this group [112,113]. A sociological study from the University of Alicante found that studying nursing does not serve as a protective factor against risky sexual behaviors [114].

Alarmingly, only 26% of students report having received formal training in sexual health during university, and just 8.9% say they would seek guidance from a healthcare professional regarding sexual matters—even though nurses play a key role in sexual health promotion and STI prevention across the life span [78].

A review of course catalogs from Spanish public universities found that only 18 out of 42 institutions offered a dedicated course in sexual education. Of these, 11 were mandatory, 2 were foundational, and 5 were elective [31,32,33,34,35,36,37,38,39,40,41,42,43,44,45,46,47,48,49,50,51,52,53,54,55,56,57,58,59,60,61,62,63,64,65,66,67,68,69,70,71,72,73,74,75,76].

In other institutions, sexual health content is integrated within broader courses such as Obstetric and Gynecological Nursing or Maternal Nursing (13 universities), Life Cycle Nursing or Pediatric Nursing (6 universities), Gender, Women’s Health, and Transcultural Health (4 universities), or Adult Nursing (2 universities). Only one university—the University of Burgos—offered an elective specifically on Health and Gender [65]. In most others, categorized sexual health topics are subsumed within core or required coursework.

Regarding course content, most programs focus on women’s reproductive health, introductory concepts of sexuality, and limited STI-related content. Overall, sexual health content remains limited and insufficiently developed [2,28,100]. It is essential to raise two key questions in order to advance toward a more equitable and effective sexual health education.

The absence of a unified national framework has led to significant disparities between universities and, more broadly, among autonomous communities, ultimately compromising the quality of education and the clinical preparedness of future healthcare professionals. These differences are not solely the result of academic or institutional choices, but also reflect the influence of historical, political, and religious factors that vary considerably by region. In more conservative regions, resistance may limit the inclusion of topics such as sexual diversity, reproductive rights, or preventive strategies against sexual violence, thus restricting curricular scope and depth.

To address these challenges, the establishment of national minimum standards would ensure that all students, regardless of location, receive comprehensive, up-to-date, and ideologically neutral education. Similarly, the implementation of faculty development programs is crucial to update scientific knowledge and strengthen pedagogical and communication skills. Such programs would enable educators to address sensitive topics with accuracy, empathy, and a critical perspective. Bridging these gaps would not only enhance clinical competencies in sexual health but also promote the development of a more inclusive, professional, and equity-driven academic environment [28,63,100].

The considerable heterogeneity among Spanish universities highlights the urgent need for a regulatory approach that ensures standardized, high-quality education for all nursing students. Minimum standards would reduce knowledge disparities and guarantee that graduates possess the clinical, ethical, and social competencies necessary for addressing sexual health. Such a framework could draw from successful international models, including those in the Netherlands and Canada, where sexuality education is fully integrated into curricula through a comprehensive, rights-based approach [115,116,117,118,119].

Several studies indicate that healthcare professionals rarely engage in peer discussions about STI prevention strategies [92,93,98,115]. Despite widespread concerns about inadequate training, up to 87.6% of professionals recognize their responsibility in delivering sexual health education and care [93,117]. A study by García Ruiz et al. concluded that nurses with appropriate knowledge of STI symptoms and current clinical guidelines can significantly contribute to reducing STI prevalence [82,116,117].

Nevertheless, many educators lack specific and updated training in human sexuality, limiting their ability to teach safely and effectively. Faculty development programs should therefore be implemented to improve clinical knowledge, enhance teaching skills, and minimize the impact of cultural taboos or stereotypes. These programs could also include training in participatory methodologies, empathetic communication, and the use of digital tools to simulate clinical encounters related to sexual health.

Although Spanish nursing curricula have increasingly incorporated sexual health content, significant institutional variability remains. Compared to other European or OECD countries, Spanish programs often adopt a less integrated and cross-disciplinary approach [118]. In contrast, countries such as the Netherlands, Sweden, and Canada explicitly include sexual health within their curricula, addressing it not only from a biomedical standpoint but also through a focus on human rights, gender and sexual diversity, sexual violence prevention, and patient-centered communication [119,120].

Nevertheless, other research shows that many professionals remain insufficiently informed about the specific characteristics of these diseases [92,121]. Sexuality, as an inherent dimension of human experience, should be approached holistically in healthcare. While 60% of healthcare personnel agree that a holistic perspective is essential, only 6% reported proactively initiating discussions about sexual health with their patients [92,104,121].

Despite these global advancements, healthcare professionals in Spain still report limited collaboration around STI prevention in clinical practice [92,93,98,115]. This is compounded by the persistent lack of specialized training in sexual health, with many professionals feeling unprepared despite acknowledging their role [93,117]. García Ruiz et al. [82,116,117] emphasize that continuous education and updated clinical guidelines can empower nurses to address STIs more effectively.

However, other studies show that healthcare professionals remain inadequately informed about STI-specific characteristics [92,121]. Sexuality, as a core dimension of the human experience, should be approached holistically in healthcare. Although 60% of professionals agree on the need for a holistic approach, only 6% report proactively initiating conversations about sexual health with patients [92,104,118].

Based on these findings, further research should evaluate the effectiveness of targeted educational interventions, examining their impact on the clinical, communicative, and attitudinal competencies of nursing students. Longitudinal studies would be especially valuable for assessing the sustained outcomes of such training in both academic and clinical settings [77,78,79,80].

In addition, it is crucial to explore the perceptions and experiences of students and faculty, identifying structural or ideological barriers to teaching sexual health. Studies could address factors such as institutional openness, faculty training, the influence of cultural taboos, and students’ willingness to engage with these topics in clinical environments. Qualitative methods—such as interviews, focus groups, or narrative inquiry—are well-suited to capturing meaningful insights that can inform the development of more effective and context-sensitive pedagogical approaches.

A particularly promising area of research involves the use of clinical simulations with standardized patients to assess students’ ability to engage in sexual health conversations with empathy, accuracy, and cultural sensitivity. These simulations foster experiential learning while providing measurable data on communication skills, decision-making, and professional confidence.

This study provides a novel contribution by offering the first national synthesis of sexual health education content in Spanish nursing curricula, analyzed through the DSMC framework. Unlike previous mapping reviews or scoping analyses that merely catalog the presence or absence of content, this work applies a structured analytical lens to assess the depth, breadth, and international alignment of sexual health education. It systematically evaluates curricular content in relation to the WHO and UNESCO guidelines, which advocate for a comprehensive, rights-based, life-course approach. By identifying institutional disparities, regional differences, and structural limitations, this study addresses a critical knowledge gap and lays the groundwork for future policy reforms, curricular development, and teacher training programs in sexual health education in Spain [4,6,7,8,119].

It is important to note that this analysis is limited to undergraduate nursing curricula and does not encompass postgraduate programs, continuing education, or other healthcare training pathways.

Finally, an emerging field of interest concerns the role of digital technologies—such as learning platforms, virtual simulations, interactive e-learning, and augmented reality—in enhancing sexual health education. These tools may help to overcome constraints related to time, space, or cultural resistance, offering safe environments in which students can practice communication and clinical skills before interacting with real patients.

### Methodological Limitations

This scoping review acknowledges a degree of subjectivity in the selection and analysis of documents, which may affect the interpretation of findings. Furthermore, the exclusion of sources without full-text access may limit the breadth of coverage. Nonetheless, the applied strategy and the diversity of consulted sources provide a comprehensive and updated overview of the current state of knowledge regarding sexual health in nursing education in Spain.

Future research should include multicenter studies to more representatively evaluate the knowledge and attitudes toward sexual health among both nursing students and professionals. It is also recommended to design and assess innovative educational interventions within academic programs. Longitudinal studies are essential to monitor the evolution of sexual health competencies throughout training and professional practice. Further research should explore perceived barriers to addressing sexuality in clinical settings and assess the integration and effectiveness of sexual health content in university curricula. Lastly, linking sexual health education to public health indicators would help to measure its impact on STI prevention and the promotion of safe sexual practices.

## 5. Conclusions

Sexual education in Spain is supported by Article 27 of the Spanish Constitution, which recognizes the right to education. However, there is currently no specific legal provision mandating the inclusion of sexual education in formal educational curricula.

Despite the robust body of scientific evidence demonstrating the effectiveness of sexual education in reducing the most severe forms of sexually transmitted infections (STIs), epidemiological data continue to show a rising incidence of these conditions. Numerous studies reveal a significant lack of knowledge among students regarding STIs and contraceptive methods. In fact, it has been reported that up to 45.6% of students engage in the practice of withdrawal (coitus interruptus), a highly unreliable method associated with a considerable risk of infection.

Furthermore, healthcare professionals often report feeling unprepared to address sexual health issues with their patients, and many express embarrassment or discomfort when doing so. Alarmingly, some nursing professionals admit to not fully understanding the transmission mechanisms of certain STIs or the key behaviors that constitute risk, highlighting critical gaps in their training.

Given this context, it is imperative that healthcare professionals reflect on the urgent need to revise and reformulate the formal educational programs aimed at training future nurses. These programs must ensure that graduates are equipped with the necessary competencies to provide effective, responsible, and evidence-based care in the domain of sexual health.

Ultimately, education remains the most powerful tool for reducing STI incidence, improving overall health outcomes, and ensuring a comprehensive approach to health within both the university population and society at large.

## Figures and Tables

**Table 1:** Thematic blocks related to search equations.

Databases	Search Strategy	Inclusion Criteria	Extracted Points	References
PubMed	Education AND Sexuality	Date range: 2015–2025	Formal education in Spain	[24,25,26,27,28,29,30]
Dialnet	Education AND Sexuality	Public universities	Nursing students and sexually transmitted diseases and training programs	[31,32,33,34,35,36,37,38,39,40,41,42,43,44,45,46,47,48,49,50,51,52,53,54,55,56,57,58,59,60,61,62,63,64,65,66,67,68,69,70,71,72,73,74,75]
SciELO	Education AND Sexuality	Full text	Inclusion of sexuality in the nursing degree curriculum	[76,77,78,79,80,81,82,83,84]
Cochrane	Education AND Sexuality		Sexuality or sexual health training of nursing degree students	[84,85,86]
Scopus	Education AND Sexuality		Knowledge of health professionals in sexual health or education	[86,87,88,89,90,91,92,93,94,95,96,97,98,99]

Source: Authors’ own elaboration.

**Table 2:** Competencies in the nursing degree in Spain.

Specific Competencies	Transversal Competencies	Core Competencies
Knowledge and skills specific to the degree program	Responsibility and autonomy related to individual, collective skills, and methods	Defined by each university
Comprehensive Nursing Care	“Learning to learn”	Acquisition of a second foreign language at B1 level
Guide, assess, and provide care	Solve complex nursing problems	Proper use of Information and Communication Technologies (ICT)
Understand health from a biopsychosocial perspective	Critical and innovative thinking based on reasoning and creativity.	Oral and written comprehension
Identify health problems	Self-management, responsibility, and dynamism	Professional ethics and deontology
	Teamwork	Promotion of good health
	Communication and information skills	

Source: Authors’ own elaboration.

**Table 3:** Sexual education in the training programs of the nursing degree at public universities in Spain.

Autonomous Community	University	Type	Syllabus Content Related to Sexuality
Community of Madrid	University of Alcalá [31]	Compulsory	I. Concepts of sexuality, family planning. II. Sexually transmitted infections (STIs).
	Autonomous University of Madrid [32]	Elective	Course syllabus not available.
	Rey Juan Carlos University [33]	Compulsory	I. Sexuality, fertility, family planning.
	Complutense University of Madrid [34]	Compulsory	I. Infertility. II. Contraception.
Valencian Community	University of Alicante [35]	Compulsory	I. Concepts of sexual and reproductive health. II. Overview of sexual and reproductive health programs, STIs. III. Clinical practicum applying prior knowledge.
	University of Valencia [36]	Basic / Compulsory	I. Sexuality and reproduction from a gender perspective. II. STIs, human sexual response, sexual dysfunctions, fertility, contraceptive methods.
	Jaume I University [37]	Compulsory	I. Sexually transmitted infections.
Andalusia	University of Huelva [38]	Basic	I. Human sexuality. II. Gender and sexuality. III. Sexual behaviors and health. IV. Sex, gender, and violence.
	University of Seville [39]	Compulsory	Course syllabus not available.
	University of Cádiz [40]	Compulsory	I. Sexual and reproductive health in adults and adolescents. II. STIs. III. Family planning and voluntary pregnancy termination.
	University of Almería [41]	Compulsory	I. Sexually transmitted infections including HPV.
	University of Granada [42]	Elective	I. Sexuality and sexual dysfunctions. II. Sexuality in illness and disability. III. Fertility regulation and intervention methods.
	University of Málaga [43]	Basic	I. Concepts of gender and health; gender-focused health policies. II. Gender-based violence. III. Sexuality and its care. IV. Sexual development across the lifespan.
	University of Jaén [44]	Compulsory	I. Sexual and reproductive health concepts. II. Anatomy and physiology. III. Nursing care in sexuality. IV. Contraceptive methods and family planning. V. STIs. VI. Gender-specific health issues.
	University of Córdoba [45]	Compulsory	I. Introduction to sexual and reproductive health. II. Family planning and contraception. III. Human sexuality and STIs.
Catalonia	University of Barcelona [46]	Compulsory	I. Introduction to sexual and reproductive health, anatomy and physiology. II. Human sexuality, family planning, health education in adolescence and climacteric. III. STIs and female genital disorders.
	Autonomous University of Barcelona [47]	Compulsory	Includes a dedicated module on sexual health.
	University of Lleida [48]	Compulsory	I. Introduction to sexual and reproductive health. II. Reproductive pathologies, STIs, gender-based violence, female genital mutilation.
	Rovira i Virgili University [49]	Compulsory	I. Sexuality in adolescents and STI prevention. II. Contraceptive methods and sexual and reproductive health.
	Pompeu Fabra University [50]	Compulsory	Includes a module on family planning and nursing care for patients with infectious diseases including STIs.
Murcia	University of Murcia [51]	Compulsory	I. Sexual and reproductive health. II. Family planning. III. Fertility and infertility. IV. Sexuality in the puerperium. V. Gynecological issues. VI. STIs. VII. Family planning reiteration.
Galicia	University of A Coruña [52]	Compulsory	I. Human sexual response. II. Fertility control. III. Workshops on sexuality and self-awareness.
	University of Ourense [53]	Compulsory	I. STIs and human sexuality. II-IV. Seminars on sexual health assessment, STI prevention, and family planning.
	University of Santiago de Compostela [54]	Compulsory	I. Reproductive health and human reproduction.
	University of Vigo [55]	Compulsory	I. Family planning and contraception. II. Sexuality during pregnancy. III. Reproductive disorders including infections.
Cantabria	University of Cantabria [56]	Compulsory	I. Sexual education. II. Workshops on family planning and contraceptive selection criteria. III. Gender-based violence. IV. Sexual and reproductive health rights.
Extremadura	University of Extremadura [57]	Compulsory	I. Gender and health, gender-based violence, sexual assault. II. Women’s health education, prevention of unintended pregnancy, and reproductive health.
Navarra	University of Navarra [58]	Compulsory	I. Sexuality-reproduction patterns. II. Fertility and its disorders in men and women. III. Risky sexual behaviors, STI prevention, gynecological pathologies.
Canary Islands	University of La Laguna [59]	Compulsory	I. Human reproduction. II. Sexual health care in adolescence. III. Lifespan female sexuality. IV. Family planning and contraceptive methods.
	University of Las Palmas de Gran Canaria [60]	Compulsory	I. Women’s sexual and reproductive health, infertility, contraceptive methods. II. Care plans for patients with sexual and reproductive health problems.
Basque Country	University of the Basque Country [61]	Compulsory	I. Infertility. II. Gynecological disorders. III. STIs.
Castile and León	University of Salamanca [62]	Compulsory	I. Diseases of the female reproductive system, HPV. II. Family planning and contraception.
	University of Valladolid [63]	Compulsory	I. Sex, gender, and sexual identity. II. Physiology and experiences of sexuality. III. Family planning, contraception, sexual education. IV. STIs.
	University of León [64]	Basic	I. Sexual harassment and assault. II. Orientation and care for sexual health.
	University of Burgos [65]	Compulsory/Elective	I. Sexual and reproductive health, contraception, infertility, STI workshops. II. Gender perspective and gender violence.
Balearic Islands	University of the Balearic Islands [66]	Compulsory	Course syllabus not available.
Aragon	University of Zaragoza [67]	Compulsory	I. Sexuality and family planning.
	University of La Rioja [68]	Compulsory	I. Reproductive cycle.
Asturias	University of Oviedo [69]	Compulsory	I. Female reproduction, fertility, contraception, STIs.
Ceuta	University of Granada [70]	Elective	I. Sexuality and sexual dysfunctions. II. Sexuality in illness and disability. III. Fertility regulation and intervention methods.
Melilla	University of Granada [71]	Elective	Same content as Ceuta campus.
Castile-La Mancha	University of Castilla-La Mancha:		
Campus of Albacete [72]	Mental health, development, and sexuality	Elective	I. Psychosocial perspective on sexuality; sexual education in health care.
Campus of Ciudad Real [73]	Maternal nursing	Compulsory	I. Infertility and nursing care during reproductive stage.
Campus of Cuenca [74]	Maternal nursing	Compulsory	I. Reproductive health.
Campus of Toledo [75]	Maternal and pediatric nursing	Compulsory	I. Infertility. II. Reproductive health. III. Health issues throughout the lifespan.

Source: Authors’ own elaboration.

## Data Availability

No new data were created or analyzed in this study. Data sharing is not applicable to this article.

## Abbreviations

The following abbreviations are used in this manuscript:

WHO	World Health Organization
STI	Sexually Transmitted Infections
HPV	Human Papillomavirus
ECDC	European Centre for Disease Prevention and Control
NND	Notifiable Disease
RENAVE	National Network for Epidemiological Surveillance
HIV	Human Immunodeficiency Virus
AIDS	Acquired Immunodeficiency Syndrome
DSMC	Dialectical Structural Model of Care
FU	Functional Unit
FF	Functional Framework
FE	Functional Element
LOMLOE	Organic Law Modifying the Organic Law on Education
UNESCO	United Nations Educational, Scientific and Cultural Organization
ICT	Information and Communication Technologies
STD	Sexually Transmitted Disease
